# What Drives Households’ Payment for Waste Disposal and Recycling Behaviours? Empirical Evidence from South Africa’s General Household Survey

**DOI:** 10.3390/ijerph17197188

**Published:** 2020-10-01

**Authors:** Abiodun Olusola Omotayo, Abeeb Babatunde Omotoso, Adebola Saidat Daud, Adebayo Isaiah Ogunniyi, Kehinde Oluseyi Olagunju

**Affiliations:** 1 Food Security and Safety Niche Area, Faculty of Natural and Agricultural Sciences, North-West University, Private Bag X2046, Mmabatho 2745, South Africa; 2 Oyo State College of Agriculture and Technology, P.M.B. 10, Igboora, Oyo State, Nigeria; omotosoabeebtunde@yahoo.com (A.B.O.); saidatdaud@gmail.com (A.S.D.); 3 International Food Policy Research Institute (IFPRI), Oro-Ago Crescent Garki II, Abuja 901101, Nigeria; A.Ogunniyi@cgiar.org; 4 Economics Research Branch, Agri-Food and Biosciences Institute (AFBI), 18a Newforge Lane, Belfast BT9 5PX, UK; kehinde-oluseyi.olagunju@afbini.gov.uk

**Keywords:** environmental safety, recycling behaviour, refuse disposal, Bivariate Probit model, South Africa

## Abstract

Safeguarding the environment and its citizens’ health remains one of the key policy priorities of the governments of many developing and emerging countries. Using the 2017 General Household Survey (GHS) dataset, this study examines the driving factors affecting households’ recycling behaviour and payment for waste disposal in South Africa. The methods of data analysis were based on descriptive statistics and a Bivariate Probit regression model. The descriptive statistics results indicate that there are 56.29% male-headed and 43.71% female headed households, with an average age of 49 years. In addition, the study shows that 89.97% of household heads had formal education with a mean monthly income of 11,099.07 ZAR/650.504 USD. The study also revealed that 22% of the households sampled had access to social grants. The results from the Bivariate Probit regression model show that household’s income, access to social grants, formal educational attainment and the age of the household were significant (*p* < 0.01) driving factors affecting households’ recycling behaviour and payment for waste disposal. The study concludes that the households’ socio-economic factors affect their recycling behaviour and willingness to pay for waste management in South Africa. Actions targeted at poverty alleviation and environmental sensitization programmes are key for facilitating environmental conservation behaviours of households in South Africa in order to achieve the environmental sustainability Sustainable Development Goal (SDG) target of the United Nations.

## 1. Introduction

The increasing volume and complexity of the waste associated with the modern economy poses a serious risk to ecosystems and human health. Globally, the amount of municipal solid waste generated is growing faster than the rate of urbanization [1]. A recent report estimates that approximately 2.01 billion tonnes of municipal solid waste is generated annually across the globe, with at least 33% not being managed in an environmentally sustainable manner [2,3,4]. The waste generated per person per day averages 0.74 kg but ranges widely, from 0.11 to 4.54 kg. Global waste is expected to grow to 3.40 billion tonnes by 2050, translating into twice the estimate of the expected population growth over the same period [5]. Waste management concerns require attention across the globe, given that waste generation and income levels are positively correlated, with evidence having established that household disposable income, on average, will continue to increase [6,7,8,9,10]. In developing countries, waste management requires a large expenditure of 30 to 50% of municipal operational budgets to collect and manage [9,11], although most cities collect only half of the waste generated [1,12,13]. In sub-Saharan Africa, approximately 62 million tons is generated per year, ranging from 0.09 to 3.0 kg per person per day, with an average of 0.65 kg/capita/day [14,15].

South Africa generates millions of tonnes of waste per year from industries, businesses and households. According to the 2019 South African Department of Environmental Affairs and Tourism (DEAT) State of Environment report, over 42 million cubic metres of general waste is generated annually, with the largest contributor (42%) being Gauteng Province [16]. In addition, over five million cubic metres of hazardous waste was reported to be produced annually in KwaZulu-Natal and Mpumalanga Provinces due to mining activities and the fertilizer production industry. The average amount of waste generated per individual per day was 2 kg, which is the highest generated waste estimate per person in sub-Saharan Africa after that for the Seychelles and Comoros, at 2.98 kg and 2.23 kg per capita per day, respectively [17]. However, the biggest contributor to the solid waste stream is mining (72.3%), followed by pulverized fuel ash (6.7%), agricultural waste (6.1%), urban waste (4.5%) and sewage sludge (3.6%) [18].

Against this background, it is clear that South Africa has not been left out of the global trend in environmental degradation through a long phase of decay, which has become particularly grave in the last decades [19]. Some definitive constitutional actions have been taken to ensure environmental conservation and safety for every citizen, regardless of their socio-economic, ethnic, political or racial status [20]. For instance, the South African government identified in the late 1990s the need to develop pollution and waste information systems (WIS) to support the implementation of waste reduction measures and effective integrated waste management [21]. However, for information systems to be sustainable, the underlying motivations or needs of key stakeholders must be understood [20,22,23]. In this regard, questions arise such as (1) how the governments’ needs can direct or shape the development of a sustainable waste information system and (2) how an information system can support effective integrated waste management in South Africa.

Furthermore, it has been observed that the collection of recyclable materials in South Africa occurs mainly through private entrepreneurs and agents for different recycling companies (DEAT, 1996). The lack of a general trend in environmental sanitation is indicative of the market-driven nature of the recycling industry, which remains vulnerable and heavily dependent on the availability of markets for recyclables (DEAT, 1996). Environmental safety was at the centre of the 1998 National Environmental Management Act (NEMA), which emphasized the need for waste avoidance [24,25,26]. The South African Department of Environmental Affairs and Tourism (DEAT) also introduced the concept of the waste hierarchy (Reduce–Reuse–Recover–Dispose) into the environmental legislation as the only possible road towards sustainable development (National Environmental Management Act No. 107, 1998) [27].

It is within the waste hierarchy that its minimization emerges as a tool to integrate reduction, reuse and recovery through recycling. Whereas waste minimization focuses on reducing the amount of waste generated, the concept of Zero Waste goes further [27], and most of the recycling is conducted by the packaging industry [28]. Furthermore, in 2000, the Local Government Municipal Systems Act (No. 32, 2000) was enacted to address the imbalance in waste disposal services. The Act requires that municipalities strive to ensure that services are provided to local communities in a financially and environmentally sustainable manner, to which there is equitable access [27].

Awareness about the environmental safety goal of adequate waste management is growing in many cities in South Africa, which is related to the United Nations’ Sustainable Development Goals (SDGs) 6 (clean water and sanitation) and 11 (sustainable cities and communities) [29,30]. The need to ensure environmental safety in rapidly urbanizing societies, such as South Africa, has come under scrutiny. The inability to ensure adequate waste management results in some cities gradually being subsumed inside the end products of their domestic and economic activities [31,32,33]. However, solid wastes can also be a blessing in disguise for households if they explore opportunities for recycling such “waste” for economic advantages. It is therefore important to explore the factors that influence households’ recycling behaviour and affect their willingness to pay for waste disposal. This is relevant for designing the “best” approach that will not only ensure efficient waste disposal strategies but also enhance households’ participation in the whole process [34,35]. 

This study aimed to examine the factors that affect households’ recycling behaviour and their willingness to pay for waste management in South Africa. The study rationale is the increasing levels of illegal waste disposal in the country, an issue that is of environmental concern, which results in an increase in pollution that can be minimized if there are regular arrangements to dispose of such solid waste. In light of South Africa’s efforts to fulfil SDGs 6 and 11, promoting waste recycling initiatives has become a key priority in the country’s environmental management strategies for reducing waste accumulation and its consequences. Despite the importance of this subject for environment and health management in the country, little attention has been devoted in the literature to examining the factors influencing households’ solid waste disposal and recycling behaviour in South Africa. The study findings will help to identify the constraints associated with households’ waste disposal and recycling practices in order to enhance the effective design of holistic programmes that can facilitate the successful implementation of policies that are required to address existing environmental challenges in South Africa and other developing and transition economies.

## 2. Methodology

### 2.1. Study Area

**Figure 1:**
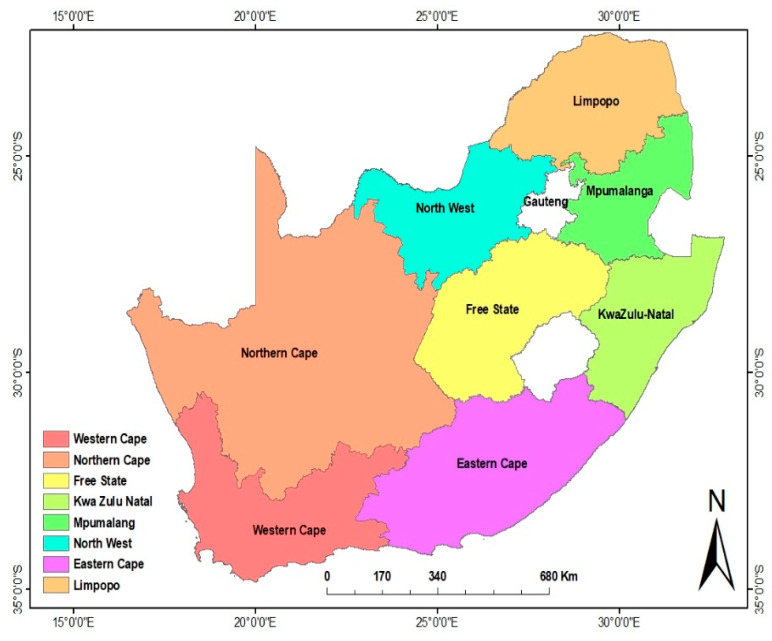
Map of South Africa, showing the different provinces of the country.

South Africa is one of the largest countries in Africa, with a population of almost 60 million people and total land area of 1,220,813 km^2^ (471,359 square mile). It has nine provinces that are rich in diverse natural resources, which determines the major occupation of the inhabitants (Figure 1). It has five large urban centres and many smaller cities and towns, all of which have municipal waste management services, which operate with various levels of efficacy. It also has many formal and informal residential areas that have varying levels of service delivery, such as the provision of potable water and refuse removal. Many people rely on social grants as an important source of income, as well as pensions, disability and child support payments [36]. 

South Africa is classified as a growing economy by the World Bank [37] and has the second biggest economy in Africa with a gross domestic product (GDP) per capita, as of 2012, estimated at 11,750 USD [38]. Approximately one-quarter of the adult population are unemployed, with many affected by poverty, living on less than 1.25 USD a day. South Africa is fast becoming a throwaway society, with a growing solid waste disposal problem. A study found that there were large quantities of empty cans, papers, tissues, polythene bags and bottles not finding their way into landfill sites, and littering streets and natural areas [39]. More than 90% of all the waste generated in South African urban areas is dumped on land not identified for its disposal. According to Darkoh [39], more than 100 million ZAR is spent yearly in South Africa to collect approximately 200,000 tons of litter that has been dumped indiscriminately. In addition, industries and businesses dispose of their wastes in open spaces, and despite the existence of laws prohibiting this activity, enforcement is severely lacking. 

### 2.2. Data and Sampling Procedures

The data utilized in this study were obtained from the 2017 South Africa General Household Survey (GHS), a nationally conducted survey of a sample of homes in the country that is performed annually. The areas covered by the GHS dataset include agriculture and food security, education and social development, environment and health, housing, and household access to services and facilities. A total of 21,225 households across the country were sampled in 2017, which constitute the pooled data analysed in this paper. The dataset also contains information on the waste disposal methods, environmental problems identified, willingness to pay for waste disposal and the recycling or selling of waste. It was compiled based on a stratified two-stage design, the first being from all nine provinces in South Africa, and the second being from the household heads. Sample weights were applied to the dataset to ensure its national representativeness [40].

### 2.3. The Theory of Planned Behaviour

The Theory of Planned Behaviour (TPB) contends that behaviour can be predicated by people’s intentions, which is determined by attitudes and behaviour intentions, which are affected by social norms and the level of controls [33,34,41,42]. It is based on the assumption that rational thinking influences people’s decisions, choices and actions, which are determined by three things: attitudes, subjective norms and perceived behavioural control. Attitudes refer to the perceived benefits, which are influenced by taking into account the consequences of an action. Subjective norms refer to people being influenced by what others think or the social attitudes to an action of family, friends and society at large [43]. The perceived behavioural controls are the factors that affect their execution of an action or behaviour, with internal control relating to their knowledge, skills and ability, and external control being the opinions and actions of others that may enable or prevent a person from taking an action and affect the time it may require. These three factors collectively result in an intention to act (or not to act) and a resulting behaviour, with the intention not necessarily being the same as the final action, depending on the factors that have influenced the intervening decisions [43,44,45]. The Theory of Planned Behaviour shows the various factors that can influence one’s behaviour. The theory states that one’s intentions are the best drivers of one’s behaviour. The TPB maintains what the Theory of Reasoned Action (TRA) postulates about human behaviour being governed by one’s attitudes and behavioural intentions characterized by the presence of social norms and the exercise of volitional control.

#### Core Assumptions of TPB

**Figure 2:**
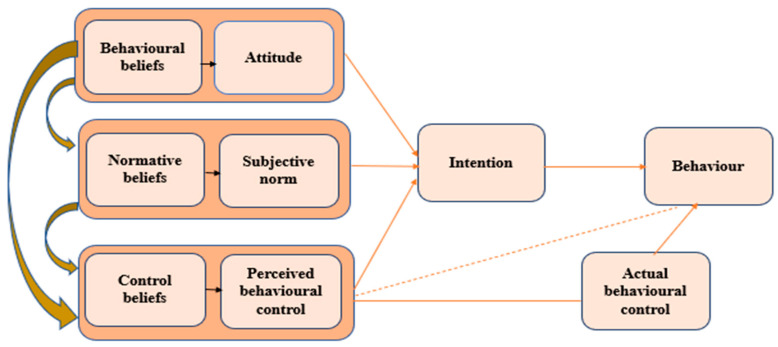
Theory of Planned behaviour (TPB).

Rational thinking finally enters the picture in this theory where it, when employed, results in rational considerations that, in turn, influence and govern the choices, decisions and behaviours of an individual [44,45]. The Theory of Planned Behaviour upholds the key assumptions contained in the Theory of Reasoned Action, with certain modifications of its own (see Figure 2).

1. The intentions of the individual largely reflects their personal attitudes or their perception of the extent of favourability of an act. This will also be influenced by their perceived and cognitive beliefs about the act [42].
2. Again, just like in the TRA, the subjective norms that the individual is exposed or privy to will also have an impact on their intentions. This is in recognition of man being, by nature, a social creature, so they will no doubt care about what others think or believe. More often than not, if society demonstrates general favourability towards an act, it is highly likely that the individual will think the same, his intentions largely shaped by the extent of approval (and disapproval) by family, friends, co-workers or pretty much any person he trusts [42,45].
3. The intentions and the resulting behaviours of the individual are affected by their **perceived behavioural control**, or what they think and believe to be their ability to actually perform or engage in the said behaviours [46]. Succeeding literature on the TPB led to the identification of the two facets of this perceived behavioural control:
  ➢ **Internal control:** This is basically what the individual perceives their own control to be like. It focuses on how the individual sees themself as being in control when it comes to performing the specific behaviour in question, and this mostly has a lot to do with the sufficiency of their knowledge, skills and abilities, and the amount of discipline they wield while performing the behaviour [42,46].
  ➢ **External control:** Other external factors also have a way of shaping how an individual behaves [42,46]. For example, the acceptance or approval of family, friends and peers is likely to influence a person into developing a positive attitude toward a behaviour, bolstering their intention to see the specific action through to the end. Time is also another factor that is external but will no doubt impact one’s level of behavioural control [44].

The TPB is more cognizant of how it is highly probable for one’s intention to be completely different from behaviour that is deliberately planned and carried out. This is mostly traced to the divergence of the level of perceived behavioural control from that of the actual control employed. [42,46].

### 2.4. Planned Behaviour Theory and the Hypothetical Framework

**Figure 3:**
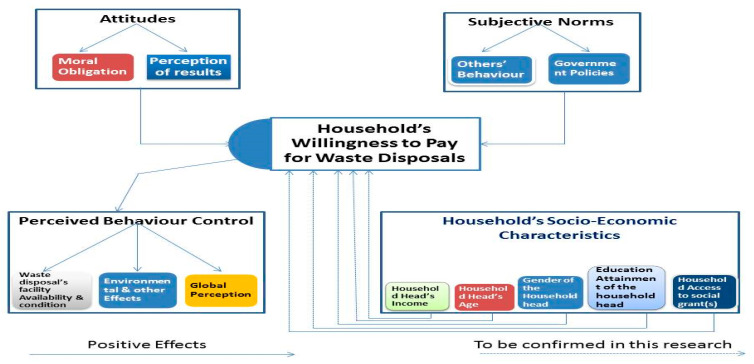
The hypothetical framework of the determinants of households’ recycling behaviour and payment for waste disposal.

The household’s waste recycling behaviour and willingness to pay for waste disposal is likely to be influenced by various factors, such as environmental factors, the availability of waste disposal facilities and their socio-economic characteristics. To understand which factors may be influential, the planned behaviour theory was used, this being a function of the theory of reasoned action [44,45]. Figure 3 depicts the theory in relation to a household’s socio-economic characteristics in structural diagrammatic form. According to the theory of planned behaviour, behavioural intentions are influenced by three components, these being perceived behavioural control, subjective norms and attitudes, whereas the variables are still subjected to change in different situations and behavioural research [41,42]. These explain the proportional change in human behaviours as a result of different behavioural intentions coupled with perceived behavioural control [42].

Within the context of this theory, the disposal behaviour of a household can be affected by the availability of refuse bins, the volumes of waste material, time factors and the perceived benefits of the final outcome. Studies by various researchers [31,32] have not included the relationship between demographic variables—such as age, gender or educational level—and households’ willingness-to-pay behaviours, with limited data being available for South Africa. This study therefore explored the relationship between people and their demographic data to develop a theoretical framework as identified in Figure 3. For the purposes of this study, variables for each component have been included that relate specifically to this study and include a fourth component of the socio-economic characteristics of the household head, as provided for in the 2017 South Africa General Household Survey (GHS) database. 

Attitudes refer to their moral obligation to care for their surroundings and health by disposing of their waste and not keeping it near them. They also relate to their perceptions of the anticipated outcomes or results of disposing of their waste. The subjective norms relate to how other people are disposing of their waste and any government policies that they feel may or may not affect them. The perceived behavioural controls relate to whether or not they have access to waste disposal facilities and what condition they are in, be it formal or informal, their surrounding environmental factors and the perceptions. The socio-demographic data available in the database of relevance to this study were the household head age, gender, education level and income, as well as their access to social grants. 

## 3. Analytical Framework and Estimation Strategy

### 3.1. Analytical Framework

The analytical framework employed for this study is based on a utility maximization framework proposed by [47,48]. This was used to derive the function for the empirical modelling of household decisions regarding the disposal of solid wastes through cash payments and recycling. Assume an economy in a county consisted of *N* identical households with utility functions represented by

Ui = U (x_i_, E_i_) (1)

where x_i_ represents the composite goods and services consumed by household i and E_i_ is the environmental quality that the household i perceives they enjoy. The environmental quality function is specified as follows:

E_i_ = h (g_i_, r_i_, d_i_) (2)

where g_i_ is the amount of waste disposed and r_i_ is the amount of materials recycled by household i. The choice of the g_i_ and r_i_ in E_i_ is also dependent on a set of demographic characteristics, d_i_. Substituting Equations (1) and (2) into (1), we rewrite the utility function as

U_i_ = U (x_i_, h (g_i_, r_i_, d_i_)) (3)

Each household maximizes utility subject to the following budget constraint:

x_i_ + p_g_g_i_ + p_r_r_i_ = m_i_ (4)

where the price of composite goods x_i_ is normalized to unity, p_g_ is the unit price of waste disposal, p_r_ is the unit price of recycling and m_i_ is the total household income. Solving the utility maximization problem yields the following demand functions for waste disposal, Equation (5), and recycling, Equation (6):

g_i_ = g (p_g_, p_r_, m_i_, d_i_) (5)

r_i_ = r (p_g_, p_r_, m_i_, d_i_) (6)

### 3.2. Empirical Estimation Strategy: Bivariate Probit Model

From the available dataset, payment for waste disposal (g_i_) was associated with Equation (5), although the dependent variable is binary in form (yes = 1, and 0 otherwise). For Equation (6), households’ recycling behaviour (r_i_) was also captured as a binary variable (yes = 1, and 0 otherwise). The choice of the binary variables for representing payment for waste disposal (g_i_) and households’ recycling behaviour (r_i_) was informed by the structure of the survey as well as a similar previous study [18]. On the basis that the dependent variables g_i_ and r_i_ are binary, it is appropriate to fit a binary regression, such as a Probit regression, to determine the recycling behaviour and payment for waste disposal. Studies such as Olagunju, et al. [49], Ogunniyi, et al. [50], Oyetunde-Usman and Olagunju [51] and [52], which had a binary dependent variable, used a Probit regression model. However, given that Equations (5) and (6) both have binary dependent variables, the errors of the two models are likely going to be correlated, hence the choice of the Bivariate Probit model, which is stated thus:

(7) $$g_{i}=\propto +\pi x_{i}+\beta_{i}k\overset{16}{\underset{k=1}{\sum }}d_{i}+u_{i}$$

(8) $$r_{i}=\omega +\varphi x_{i}+\rho_{i}k\overset{16}{\underset{k=1}{\sum }}d_{i}+z_{i}$$

where $\beta_{i}k$, $\rho_{i}k$, φ, π, ω and α are the estimated parameters. Both Equations (7) and (8) also include a set of independent variables denoted as ($d_{i}$) in dummy form and $x_{i}$ in continuous form, while $u_{i}$ and $z_{i}$ represent the error terms. In Equations (7) and (8), the necessary assumptions for the Bivariate Probit model estimation were made through the use of STATA12 statistical software, where the parameter of the correlation between the error terms in the two equations is presented. In addition, the likelihood ratio test evaluates whether the value of ρ is statistically significant (*p* < 0.05). If this is so, it confirms a significant correlation between the error terms, which further emphasizes that estimating the models separately using a conventional Probit model would give biased parameters [31].

## 4. Results and Discussion

### 4.1. Distributions of Socio-Economic Variables

The results for the socio-economic variables of the respondents across the nine provinces in South Africa (Table 1) reveal that the mean age of the household heads was 49 years, which indicates that South Africa has a dynamic household head age group, which could be economically advantageous to the nation for the workforce. The Eastern Cape had a mean age of 51.67 years (which is approximately 52 years), while Gauteng had the lowest (46.20 years, approximately 47 years). The result was in line with [10,53], whose report ascertained that most of the household heads in South Africa were in the economically active age range. The pooled data results reveal that an average (89.97%) South African had formal education. The Free State Province reported the least attainment in formal education (76.31%), and the Western Cape Province, the most (96.91%). Education plays a crucial role in households’ socio-economic status and environmental consciousness, and [54,55,56] play an important role in mitigating the adverse impact of environmental challenges on an individual’s socio-economic status.

Our results also show that the mean monthly income for households in South Africa was estimated at 11,099.07 ZAR/650.50 USD (1 USD = 17.06 South African rand), with the incomes in Gauteng and Mpumalanga Provinces being the highest on average at 16,037.47 ZAR/939.94 USD and 14,302.99 ZAR/838.28 USD, respectively; Limpopo (5508.31 ZAR/322.84 USD) and Eastern Cape Provinces (7740.40 ZAR/453.66 USD) had the lowest earning households in terms of average income levels. This result is not in conformity with a number of studies [9,33,57], which reported that there is a negative relationship between poverty (low income) and environmental conservation as indicated in the study. This relationship leads to environmental degradation. Fabra [58] reported that “there is a distinct connection between poverty status and environmental degradation in a nation, moreover, if the poverty status is not well managed, it can cause serious environmental problems”.

Furthermore, this research also revealed that out of every 10 households sampled (21.90%), two reported having access to social grants, with the Province of Gauteng and KwaZulu-Natal having the highest numbers of 49.96% and 33.37%, respectively. These grants are intended to reduce poverty in order to improve households’ environmental conservation; there is need to reduce individuals’ overdependence on degraded natural resources [2,59,60,61].

Male-headed households accounted for approximately 56.29%, while the remainder were female-headed (43.71%), which is different to the figures reported elsewhere [61,62,63,64,65]. In North West, Gauteng and Limpopo Provinces, 85.78%, 77.25% and 51.93% households, respectively, were headed by females, which could account for their being the poorest provinces, with studies having established that households headed by females tend to be poorer than those headed by males [63,66,67,68].

### 4.2. Households’ Waste Disposal Methods and Perception of Environmental Problems

The waste disposal methods adopted in a community dictate the level of environmental safety in that society. A number of authors [69,70,71,72] opined that there is a significant relationship between waste disposal methods and environmental safety due to contact with harmful and dangerous pollutants, which can be detrimental to health. Table 2 presents the various methods of waste disposal in South African provinces, the main one being a communal refuse dump/container (75.68%). Each province adopted specific methods, with disposal through local authorities/private companies (at least once a week) accounting for 70.00% and 59.11% of the households in the Western Cape and KwaZulu-Natal, respectively. Free State (10.21%) and Mpumalanga (11.55%) Provinces had the least use of these means of waste disposal.

Households from Gauteng (27.74%) and Eastern Cape (10.78%) ranked highest in terms of refuse dump use. This supports Makgae [73], who reported that waste disposal and management were serious problems in South Africa, as they were poorly funded, which encouraged financially incapacitated households to adopt their own refuse disposal methods. Other problems associated with waste management discussed by Ogola, et al. [74] include inadequate waste collection services, the irrational and illegal dumping of refuse, poor waste recycling techniques and inadequate policy implementation for waste management.

Table 3 shows the prevailing environmental problems as perceived by household heads and that littering is a major environmental problem (35.75%), specifically in Mpumalanga (39.01%) and the Western Cape (33.27%). Irregularity in waste removal or no waste removal at all was the highest (23.90%) in Gauteng and of least concern in the Free State (5.93%) and Mpumalanga (8.36%). Many households perceived littering and refuse dumping anywhere as an environmental problem that requires drastic measures for its control or eradication. 

Table 3 indicates that 8.09% of the households in South Africa faced the problem of water pollution, with those in Gauteng and Western Cape having the highest percentages of 14.42% and 9.21%, respectively. This may be due to the contamination of water bodies such as streams and rivers as a result of inappropriate waste disposal. Studies by [75,76,77] revealed that drinking contaminated and polluted water could result in diseases, and that of the homes with inadequate access to potable water, approximately 20% treated their water before drinking. According to various authors [78,79,80], this calls for appropriate waste disposal to ensure access to safe and clean water for home and industrial usage.

### 4.3. Paying for Waste Disposal and Recycling Behaviour of the Respondents

Slightly over half (58.46%) of households pay for waste disposal in South Africa, with Free State and KwaZulu-Natal provinces indicating the highest values of 92.02% and 87.85%, respectively, while Mpumalanga (19.98%) and Eastern Cape (16.24%) had the lowest. Table 4 indicates the households not paying for waste disposal, with the Free State and KwaZulu-Natal having the highest values of 90.81% and 92.42%, respectively, while the Western Cape (7.46%) and Limpopo (8.85%) reported the lowest percentages. This indicates people’s attitudes favouring pro-environmental behaviour if services are offered [59].

**Figure 4:**
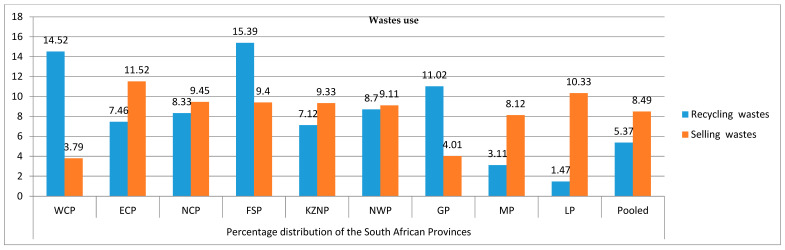
Percentage distribution of households based on recycling or selling waste.

The respondents involved in waste recycling and selling and the nature of such activities are presented in Figure 4 and Table 5, with 5.37% conducting waste recycling and 8.49% selling waste. The Free State (15.39%) and Western Cape (14.52%) had the highest proportions of their households involved, while Limpopo (1.47%) and Mpumalanga (3.11%) reported the lowest. The distribution of households in each province based on selling waste revealed that the Eastern Cape (11.52%) and Limpopo (10.33%) reported the highest proportions. Paper (4.31%) and plastic (2.01%) were the most recycled items, which is in line with existing studies on recycling [35,81,82]. Several initiatives to improve household participation in waste recycling have been initiated but encountered set-backs due to poor sustainability at both the government and household levels. These initiatives should be intensified through sensitization/awareness programmes and by providing incentives to those who undertake recycling to promote households’ involvement in the recycling of waste products.

### 4.4. Parameter Estimates for Recycling Behaviour and Payment for Waste Disposal in South Africa

Table 6 presents the results of the Bivariate Probit analysis of cash payments for waste disposal and recycling in South Africa. It is important to mention that we conducted a test for the presence of multi-collinearity among the explanatory variables in order to determine the appropriate variables to be included in the Bivariate Probit analysis. Following the test, we excluded variables that failed the collinearity test from the final model. Table 6 also shows the level of tolerance of the variables in the analysis, which also serves as a decision criterion for the choice of variables for the Bivariate Probit model. The correctness of the estimated model, that there exists a significant relationship among the selected independent variables and the dependent variables at *p* < 0.01, was established by the likelihood ratio test with a result of rho equal to zero. 

Furthermore, the Wald Chi-Square test (*p* < 0.01) attested to the goodness of fit of the model. The computed Athrho for the cash payments (0.1879, *p* < 0.01) and recycling of waste (0.1990, *p* < 0.01) shows statistical significance, which denotes the correctness of the model used. The results in Table 6 also show that household income is statistically significant (*p* < 0.01) in the Bivariate Probit model. This shows that as household income increases, the probabilities of recycling and paying for waste disposal will also increase. A similar result was reported in Olagunju, et al. [83] where the study found that willingness to pay for improved service management is influenced significantly by household income. In addition, the social grant parameter estimates in the two models were statistically and positively significant (*p* < 0.01). This implies that households that have access to social grants have higher probabilities of recycling and paying for waste disposal than their counterparts not receiving any. These results were in line with those of Guerin, et al. [84] and Omotayo, et al. [85], who ascertained that income is a major determinant factor in waste recycling behaviour. This implies that there will be an increase in a household’s likelihood of paying for environmental services if there is an increase in its income.

The formal education of the household *(p* < 0.01) and water pollution (*p* < 0.01) caused by the indiscriminate dumping of refuse have a positive significant influence on recycling behaviour and cash payments for disposing of waste. It shows the probability that as the level of education of the household head and the water pollution caused by refuse dumping near water channel increases, holding all other variables in the model constant, there will be a high tendency for waste recycling and payment for waste disposal. In addition, the age of the household head *(p* < 0.01) has a negative significant influence on recycling behaviour but a positive significant influence on the amount paid for waste disposal in the study.

The parameter estimates of littering *(p* < 0.01) and land degradation *(p* < 0.01), as forms of perceived problems associated with indiscriminate waste disposal, show a positive statistically significant relationship in the waste recycling model but a non-significant one (*p* > 0.10) in the model for payment for waste disposal. These results conform with a priori expectations and the work of Whitmarsh [86], which reported that environmental coping strategies are born out of people’s ability to understand the problems in the environment. Some province parameter estimates show statistical and positive significance (*p* < 0.01), with households in the Western and Eastern Cape showing higher probabilities of paying for waste disposal than households in other provinces. In the model estimated for paying for waste disposal, the provincial parameters with statistical significance are Western Cape, Eastern Cape and North West at *p* < 0.05.

## 5. Conclusions

Environmental sustainability remains a principal goal of the United Nations’ 2030 target. Therefore, safeguarding the environment and health of South Africa’s citizens is an issue of utmost importance, even though various levels of South African governments have implemented different waste disposal avenues for her populace; however, the inefficiency of these environmental programme(s) in many parts of the nation necessitates further efforts in the annual household data overview. Therefore, we utilized the South Africa’s General Household Survey (GHS) 2017 dataset to investigate factors influencing households’ solid waste disposal and recycling behaviour in South Africa. The descriptive statistics findings show that there are 56.29% male-headed households and 43.71% female-headed households in the study. An average age of 49 years was recorded in the study. In addition, about 89.97% of the South African household heads had formal education, with the mean monthly income estimated at 11,099.07 ZAR/650.504 USD. Moreso, our study revealed that out of every 10 households sampled (21.90%), two reported having access to social grants. The findings further identified different waste disposal methods, with the majority of households from the Western Cape and Northern Cape using communal refuse dumps/containers. Littering was a major environmental problem closely followed by irregular/no waste removal, then air pollution, land degradation and noise pollution. Many households perceived littering and the dumping of refuse anywhere as an environmental problem that requires drastic measures for its control or eradication. 

Approximately 5% and 8% of the households were recycling and selling waste, respectively, with paper and plastic constituting the main recycling materials in the Northern Cape and North West province, respectively. These findings call for sensitization/awareness programmes and the provision of incentives to enhance households’ involvement in recycling waste products. Approximately half of the respondents were paying for waste disposal, while the majority of the households not paying were willing to do so in the future, provided that the problems associated with waste disposal and recycling were identified and solutions found. The results related to the parameter estimates of waste recycling behaviour and cash payments for waste disposal revealed that they are being affected by either some or all of the socio-economic factors, namely, household income, access to social grants, formal educational level and the age of household head. Therefore, this study concludes that the socio-economic factors affect households’ recycling behaviour and willingness to pay for waste management in South Africa as proposed by our framework (Figure 3). The study therefore recommends that the government of South Africa should intensify their efforts on programmes targeted at poverty alleviation in order to improve the household’s socio-economic status as well as to foster environmental sensitization programmes through the adequate education of the citizens on the importance of sanitation for public heath, which is key to facilitating environmental conservation behaviours of households in South Africa as well as the achievement of the environmental sustainability target of the SDG goals.

## Figures and Tables

**Table 1:** Distribution of respondents by selected socio-economic characteristics of household heads.

Socio-Economic Variables	Percentage Distribution of the South African Provinces									
	WCP	ECP	NCP	FSP	KZNP	NWP	GP	MP	LP	Pooled
Age (Years)	49	52	51	49	49	49	47	48	50	49
Income (Rands)	12,167.65	7740.40	8518.34	13,614.60	9282.93	7966.12	16,037.47	14,302.99	5508.31	11,099.07
Social Grants (%)	20.91	27.75	9.25	12.39	33.37	14.24	49.96	17.48	23.73	21.90
Formal Education (%)	88.43	96.91	78.36	76.31	87.76	86.89	91.36	89.41	81.31	89.97
Gender										
Male (%)	73.23	69.17	74.20	59.76	74.27	14.22	22.75	58.47	48.07	56.29
Female (%)	26.77	30.83	25.80	40.24	25.73	85.78	77.25	41.53	51.93	43.71

Note: WCP = Western Cape Province; ECP = Eastern Cape Province; NCP = Northern Cape Province; FSP = Free State Province; KZNP = KwaZulu-Natal Province; NWP = North West Province; GP = Gauteng Province; MP = Mpumalanga Province; LP = Limpopo Province.

**Table 2:** Percentage distribution based on waste disposal methods.

Waste Disposal Methods among Households	Percentage Distribution of the South African Provinces									
	WCP	ECP	NCP	FSP	KZNP	NWP	GP	MP	LP	Pooled
Local authority/private company at least once a week	70.00	55.47	33.11	10.21	59.11	33.42	40.47	11.55	30.12	46.24
Local authority/private less often than once a week	15.11	7.24	0.14	0.00	15.17	7.06	0.16	3.21	12.91	14.11
Community members contracted	1.12	1.02	2.45	0.00	4.56	9.12	2.45	5.45	4.17	5.01
Community members contracted by the municipalities less often than once a week	7.13	3.14	5.47	7.70	8.11	9.92	2.24	0.13	5.47	9.18
Community members at least once a week	60.47	64.99	57.00	34.17	37.72	25.01	53.41	60.47	44.17	49.86
Community members at less often than once a week	1.36	2.33	8.40	3.41	0.05	4.21	4.16	1.23	3.41	28.64
Communal refuse dump/container	84.49	71.14	80.00	62.77	70.42	53.07	63.71	51.34	74.99	75.68
Own refuse dump	1.29	10.78	0.10	0.84	0.69	1.31	27.74	3.14	2.33	8.90
Dump or leave rubbish anywhere	0.07	0.13	0.42	1.15	1.14	4.22	2.24	2.33	3.41	11.83
Other places	0.42	0.40	6.07	1.27	1.26	0.14	3.11	0.78	7.24	12.31
Missing responses	0.03	0.14	2.42	2.81	1.05	0.07	2.14	0.40	6.47	10.12

WCP = Western Cape Province; ECP = Eastern Cape Province; NCP = Northern Cape Province; FSP = Free State Province; KZNP = KwaZulu Natal Province; NWP = North West Province; GP = Gauteng Province; MP = Mpumalanga Province; LP = Limpopo Province.

**Table 3:** Percentage distribution of environmental problems.

Environmental Problems	Percentage Distribution of the South African Provinces									
	WCP	ECP	NCP	FSP	KZNP	NWP	GP	MP	LP	Pooled
Irregular or no waste removal	10.01	13.27	14.42	5.93	15.96	16.81	23.90	8.36	11.35	18.86
Littering	33.27	25.71	36.52	24.03	28.42	40.21	25.82	39.01	13.31	35.70
Water pollution	9.21	8.52	3.21	4.05	7.06	5.11	14.42	5.19	4.17	8.09
Air pollution	11.06	10.11	4.02	4.19	9.12	4.12	11.33	6.64	5.08	8.94
Land degradation	10.72	11.42	4.07	5.15	17.17	6.54	22.26	9.41	15.57	14.91
Noise pollution	9.80	10.00	3.13	8.08	0.56	3.39	4.04	7.11	7.29	3.95

WCP = Western Cape Province; ECP = Eastern Cape Province; NCP = Northern Cape Province; FSP = Free State Province; KZNP = KwaZulu Natal Province; NWP = North West Province; GP = Gauteng Province; MP = Mpumalanga Province; LP = Limpopo Province.

**Table 4:** Percentage distribution based on paying and willingness to pay for waste disposal.

Willingness to Pay for Waste	Percentage Distribution of the South African Provinces									
	WCP	ECP	NCP	FSP	KZNP	NWP	GP	MP	LP	Pooled
**Paying**										
Yes	45.10	16.24	31.83	92.02	87.85	80.98	31.66	19.98	21.63	58.46
No	31.63	82.15	50.35	7.60	6.05	16.18	48.78	17.36	74.56	39.50
Do not know	27.66	0.18	16.24	0.68	1.16	2.58	18.76	0.09	1.50	0.21
Missing	0.61	1.43	1.57	0.00	4.94	0.26	0.80	63.35	2.29	1.83
**Willing to pay**										
Yes	7.46	25.68	71.28	90.81	92.42	47.23	37.10	14.68	8.85	33.21
No	29.07	56.52	4.45	8.65	6.91	22.80	43.88	20.91	51.29	31.61
Do not know	0.46	16.24	1.71	0.54	0.66	10.21	18.76	64.03	39.61	34.35
Missing	70.47	1.55	2.55	0.00	0.00	19.77	0.26	0.38	0.25	0.85

**Table 5:** Percentage distribution types of materials recycled.

Materials Being Recycled	Percentage Distribution of the South African Provinces									
	WCP	ECP	NCP	FSP	KZNP	NWP	GP	MP	LP	Pooled
Paper	8.33	4.78	9.81	10.56	8.65	2.91	0.54	2.55	0.46	4.31
Glass	1.71	0.54	0.66	0.00	4.45	1.71	0.46	1.55	0.56	1.03
Plastics	7.46	1.28	3.21	1.33	0.51	5.56	1.20	3.00	0.51	2.01
Metal	1.28	1.56	1.81	0.00	0.00	0.56	0.00	0.00	0.51	0.36
Oil	1.52	2.11	0.00	1.71	1.28	0.00	0.51	0.56	0.00	0.51

WCP = Western Cape Province; ECP = Eastern Cape Province; NCP = Northern Cape Province; FSP = Free State Province; KZNP = KwaZulu Natal Province; NWP = North West Province; GP = Gauteng Province; MP = Mpumalanga Province; LP = Limpopo Province.

**Table 6:** Bivariate Probit results for factors influencing recycling and payment for waste disposal.

Socio-Economic Variables	Household’s Recycling Behaviour			Payment for Waste Disposal			
	Coefficient	Std. Error	Prob.	Coefficient	Std. Error	Prob.	Tolerance
Income (Rands)	0.0021	0.0000	0.0000	0.0030	0.0004	0.0000	0.5341
Household head’s age (Years)	−0.0311	0.0000	0.0000	0.4532	0.0034	0.0000	0.8721
Formal education (%)	0.0216	0.0001	0.0000	0.5432	0.0267	0.0000	0.9841
Access to social grant (%)	0.0218	0.0012	0.0000	0.3139	0.0394	0.0000	0.6723
Gender (%)	−0.1179	0.0816	0.1480	0.0579	0.0124	0.0102	0.8672
Dispose of refuse anywhere (%)	0.1167	0.1211	0.1239	0.6754	−0.1243	0.0000	0.8941
Irregular or no waste removal (%)	0.2171	0.1891	0.1109	0.1229	−0.0332	0.0000	0.5561
Littering (%)	0.1121	0.0010	0.0010	0.7452	0.9812	0.1289	0.8871
Water pollution (%)	0.3218	0.0081	0.0000	0.1876	0.0182	0.0004	0.8631
Air pollution (%)	0.1891	0.1771	0.1127	0.0405	0.0037	0.0000	0.7611
Land degradation (%)	0.0114	0.0012	0.0000	0.0342	0.0457	0.1182	0.9611
Noise pollution (%)	0.2111	0.2101	0.3423	0.5341	−0.4991	0.1002	0.4155
Western Cape—WC (%)	0.5231	0.1271	0.0000	0.0034	0.0001	0.0000	0.7842
Eastern Cape—EC (%)	0.3271	0.0782	0.0001	0.0738	0.0032	0.0011	0.8790
Northern Cape—NC (%)	−0.0125	0.0421	0.3290	0.0113	0.1241	0.1209	0.7677
Free State—FS (%)	0.0212	0.1287	0.2902	0.1109	0.7320	0.4995	0.5479
North West—NW (%)	−0.2109	0.3322	0.2120	0.0220	−0.0090	0.0000	0.6682
Mpumalanga—MP (%)	0.1199	0.2331	0.6721	0.2391	−0.4590	0.3290	0.5322
Constant term	0.0728	0.0002	0.0000	0.3203	0.0311	0.0003	
AthRho	0.1871	0.0214	0.0000	0.1984	0.1990	0.0000	
Rho	0.1784	0.0067		0.1713	0.1002		
Log likelihood	−16,762.872						
Number of Observations = 21,225; chi^2^ = 78.90, Prob chi^2^ = 0.0000							
